# The Use of Rumination and Reappraisal in Adolescents Daily Life: Links to Affect and Emotion Regulation Style

**DOI:** 10.1007/s10578-021-01302-7

**Published:** 2021-12-17

**Authors:** Neus Zuzama, Josep Roman-Juan, Aina Fiol-Veny, Maria Balle

**Affiliations:** University Research Institute of Health Sciences (IUNICS), University of the Balearic Islands, Cra. de Valldemossa, km 7.5, 07122 Palma, Mallorca Spain

**Keywords:** Ecological momentary assessment, Adolescence, Emotion regulation, Positive and negative affect, Reappraisal and rumination

## Abstract

This study explored the association between temperament—i.e., positive affect (PA) and negative affect (NA)—and emotion regulation (ER), and what momentary factors influence the selection of rumination or reappraisal during adolescents’ daily life. The type of social situation in which negative events occurred, the self-rated degrees of discomfort, the types of predominant emotions experienced, and the use of reappraisal and rumination were assessed at 24 different times with an ecological momentary assessment approach given to 71 adolescents. PA, NA, and ER style were evaluated using self-reports. Bivariate Pearson correlations analysis revealed that NA and negative ER style correlated positively with the rumination use whereas PA correlated negatively with the rumination use. Negative ER style moderated the relationship between NA and the frequency with which rumination was used. The moderated function of positive ER style could not be tested due to its lack of association with the rumination use. Adolescents selected rumination more often during family-related events and when experiencing depression-like emotions. No interaction effects were shown between negative ER style and the momentary factors related with the type of social situation and the type of prevailing emotion during negative event. No associations between study variables and reappraisal were found. This study provides a better understanding of ER patterns in adolescence.

## Introduction

Adolescence is a period characterized by continuous changes at the physical, emotional, cognitive, and social levels, and these fluctuations can become a source of stress. In their everyday lives, adolescents are exposed to many stressful situations that induce specific emotions (e.g., sadness, fear, guilt, etc.) which can trigger emotion regulation (ER) and coping processes [1, 2]. ER is a process that is relevant to adaptive functioning and through which individuals influence how they experience and express emotions [3]. Failure to regulate emotions or affect is a risk factor for mental difficulties later on in life [4]. Research has shown that adolescents with poor ER, namely, emotion dysregulation, are more vulnerable to internalizing and externalizing problems [5]. Therefore, the ability to regulate emotions during everyday circumstances may play a key role in reducing the risk of psychopathological issues during this significant developmental period [2].

Research in this field suggests that variations in temperament are likely to contribute significantly to the development of a particular ER style e.g., [6] and influence the preference of certain ER strategies that adolescents use to cope with factors they perceive as stressful [7]. Clark and Watson’s [8] tripartite model stands out among the temperamental models that has been used to investigate temperamental contributions to ER in children and adolescents. In this model, there are two primary factors: positive affect (PA; the tendency to experience positive emotions, such as enthusiasm and excitement) and negative affect (NA; the tendency to experience negative emotions, such as fear, sadness, and anger). Different studies have shown that a heightened NA in children and adolescents is associated with the engagement in a dysfunctional ER-style [6] and an increased use of maladaptive ER strategies (e.g., rumination; [9]). Conversely, high levels of PA may be protective, as they attenuate emotion dysregulation [10] and favor the ability to more easily access more adaptive ER strategies (e.g., cognitive reappraisal; [11]).

Given that the way in which adolescents regulate their daily emotions ultimately affects their mental health and well-being, it is important to select appropriate ER strategies when dealing with stressful events with the goal of modifying the magnitude and/or type of individuals’ emotional experience [12]. Although the use of either of these strategies depends on context, individuals seem to have a dispositional trend or style to use one type more than the other in many situations [13, 14]. Cognitive ER strategies are usually divided into positive or adaptive (i.e., associated with beneficial long-term outcomes) and negative or maladaptive (i.e., associated with negative long-term outcomes; e.g., [15, 16]). Cognitive reappraisal and rumination have been the most studied as examples of adaptive and maladaptive ER strategies, respectively [17]. Even though both strategies aim to regulate emotions, the results derived from each one are different. Reappraisal can relieve discomfort in many situations as it consists of reframing the meaning of a situation in positive terms, which changes the person’s judgment of that situation [18]. As a result, reappraisal has a beneficial effect on affect, self-esteem, and adjustment [19] as well as causing individuals to experience more positive emotions and less negative ones [20]. On the other hand, rumination involves thinking repeatedly about a negative event or emotion [21]. As a consequence, rumination triggers more negative emotions [22], depressive symptoms [23], and fewer positive emotions [24], namely, a maladaptive result [15].

Despite recent inroads, studying the association between temperament (e.g., PA and NA) and ER in adolescents may be of particular importance as adolescence is a period during which individuals are vulnerable to the onset of mental health disorders [25]. So far, research in this field has been mainly cross-sectional, and the information provided has been limited. Such an approach is valid for examining ER as a “trait”; but other approaches are needed to uncover the nature of the ER process in individuals’ everyday lives [20]. We consider the Ecological Momentary Assessment (EMA) to be a more well-suited approach to understanding how adolescents regulate emotions in their daily lives.

At this point, the first contributions to the study of ER during adolescents’ day-to-day lives (see [26–28]) have not included the association between temperament (i.e., PA and NA), ER style, and the momentary ER strategies used to attenuate negative emotional states. Consequently, the present study puts the focus on these variables. Regarding momentary ER strategies, we focus specifically on cognitive reappraisal and rumination. Despite the substantial increase of research examining the consequences and use of ER, much less is known about what momentary factors may influence the selection of individual ER strategies. The above-mentioned studies represent the first attempts to examine the influence of some factors (e.g., the intensity and lability of different negative emotions) on adolescents’ selection of a particular ER strategy. For instance, Lennarz et al. [26] and Silk et al. [27] propose that rumination is implemented in response to high degrees of discomfort, whereas Shafir et al. [29] propose that when anticipating low intensity stimuli, the implementation of reappraisal is preferred. Nevertheless, the role that other momentary factors may play in such selections remains understudied.

During adolescence, social relationships become especially important both among peers and the family. In such interactions, negative emotions often arise and individuals try to manage them in order to maintain good relationships with their significant others. Several studies in this field have shown that different ER strategies are associated with different social outcomes. For instance, John and Gross [30] found that reappraisers are perceived by their partners as emotionally engaged and responsive, and are more frequently sought after as friends compared to non-reappraisers. Further, Lavalle and Parker [31] found that young adolescents who tend to ruminate over friendship problems are more vulnerable to jealousy around their friends -which is related to greater conflict with friends- are less well-liked by peers in general, have more mutual peer enemies, and are victimized by peers. Similarly, Jostman et al. [32] found that late adolescents who felt that their relationship quality was threatened and were prone to ruminative thoughts were less able to change their negative thoughts about their partners to positive thoughts than those who were not prone to rumination. As a whole, previous research suggests that those who are better emotion self-regulators may not only be more reliable romantic partners, but also are better at managing conflict and, consequently have higher quality friendship and romantic relationships than those who are poor emotion self-regulators [33]. As far as the family is concerned, research appears to support a bidirectional model of adolescent self-regulation development and parent-adolescent relationship quality. Adolescents with poor self-regulation abilities may be difficult for their parents to be managed, which may be associated with poor relationship quality [34]. On the other hand, adolescents with high quality relationships with parents may be able to better develop good self-regulation abilities [34]. Noteworthy, although prior studies have examined social outcomes according to the ER strategies used, the way in which different everyday situations (e.g., conflicts with family members or friends) may influence on adolescent’s selection of a particular ER strategy has not yet been examined.

Finally, the effect of specific ER strategies on certain negative emotions has been explored. Silk et al. [27], for example, reported a direct link between the use of reappraisal and a greater regulation of anger, but not sadness or anxiety. In comparison, the use of rumination was associated with less regulation of anger and sadness, but not anxiety. The outcome of the process of coping with each of these emotions contributes to determining the social interactions that occur [35]. While effective anxiety regulation is associated with a greater sense of control and autonomy, adequate anger regulation is related to a constructive style of conflict resolution and appropriate sadness regulation in positive social relationships [36]. Nonetheless, the role that certain emotions play in selecting one ER strategy or another is still unknown.

### The Current Study

Considering the above, the current study consists of an EMA with two overarching goals. First, we examined whether PA and NA (considered as temperamental traits) are related to the use of rumination and reappraisal in daily life. Further, we aimed to explore whether ER style (both positive and negative) plays a moderating role in such relationship. On the basis of the background set forth in the theoretical framework, we expected a positive association between PA and reappraisal use as well as between NA and rumination use. Conversely, we expected a negative association between PA and rumination use just as between NA and reappraisal use. Moreover, we hypothesized there would be a greater association between NA and the use of rumination in adolescents whose ER style is more negative and less positive, and between PA and the use of reappraisal in adolescents whose ER style is more positive and less negative. By contrast, we hypothesized that there would be a minor association between NA and the use of reappraisal in adolescents whose ER style is more negative and less positive, and between PA and the use of rumination in adolescents whose ER style is more positive and less negative.

Secondly, we explore the following factors stemming from the situations classified as aversive: the type of social situation in which negative events occurred, the degree of discomfort triggered by these negative events, and the type of predominant emotion that the adolescents experienced during the events. In the present study, the type of social situation could be a conflict with friends or with family, and the type of predominant emotion could be an emotion related to anxiety or depression. These factors could be involved in the selection adolescents make regarding one ER strategy or another when facing a negative emotional state. In addition, we aimed to explore whether ER style (both positive and negative) plays a moderating role in the relationship between the momentary factors discussed above and the selection of a particular ER strategy. Most of the work done regarding the second objective is exploratory since, to our knowledge, there is no previous literature that specifically addresses this topic. We only make our hypothesis based on the degree of discomfort, as this is the only variable for which there are currently data in other studies. Specifically, we expect that rumination will be implemented in response to high degrees of discomfort and reappraisal in response to low degrees of discomfort.

## Method

### Participants

Participants were extracted from a large pool of adolescent students enrolled in a 3-year research project concerning emotion regulation. At the beginning of the project, for the sample recruitment, a total of 15 from 76 high schools of the city where the study was conducted (i.e., 20% of high schools) were randomly asked to take part in the project. Six high schools rejected to participate, resulting in a total of nine high schools included.

From the nine randomly selected high schools who agreed to participate in the study, the sample initially comprised 78 adolescents. Exclusion criteria were suffering from any diagnosed psychopathological disorder [37], having been undergoing psychological or psychiatric treatment, or presenting incomplete self-report measures. As a result, two participants were excluded due to the presence of a psychopathological condition (one adolescent had major depressive disorder and another one had social anxiety disorder), and five were excluded due a lack of data derived from technological errors (*n* = 7 out of *n* = 78, 8.97%; see the Data Acquisition and Pre-processing section). Thus, the final sample in the present study was comprised of 71 adolescents (*M*_*age*_ = 14.68; *SD*_*age*_ = 0.82; range = 14–17 years old; 55.50% girls). All participants were middle-class Caucasians, and from both urban and rural areas. The study was approved by the University’s Research Ethics Committee, and all participants and their parents/legal guardians provided written consent at the beginning of the study.

### Measures

#### Psychopathological Disorders

The *Kiddie-Schedule for Affective Disorders and Schizophrenia, Present and Lifetime Version* (K-SADS-PL; [38]) is a semi-structured clinical interview designed to identify current and lifetime child psychiatric diagnoses based on the Diagnostic and Statistical Manual of Mental Disorders [37]. We used the Spanish version of the interview, developed by Ulloa et al. [39], which gathers data from both the adolescents and their parents. A diagnosis can be coded as definitive, probable (when individuals meet the diagnostic criteria of 75% or more), or absent. This interview was used to determine whether the adolescents presented any psychiatric disorders. Only participants who were suspected of suffering from a current psychiatric disorder were excluded. The presence of lifetime disorders was also recorded, but was not used as an exclusion criterion.

#### Positive and Negative Affect

The *Positive and Negative Affect Schedule for Children and Adolescents* (PANASN; [40]) is a version of the Positive and Negative Affect Schedule (PANAS; [41]). It was designed to be used with children and adolescents from 7 to 17 years of age, and the original Spanish version was employed. This self-report instrument consists of two 10-item scales measuring PA and NA. Participants were asked to rate items according to how they usually feel certain feelings (1 = never, 2 = sometimes, 3 = very often). Cronbach’s *α* for our screened sample was 0.66 for PA and 0.88 for NA.

#### Emotion Regulation Style

The *Cognitive Emotion Regulation Questionnaire* (CERQ; [13]) in its Catalan version [42] was used. This self-report instrument is designed to assess cognitive ER, defined as “the cognitive way of managing the intake of emotionally arousing information” (p. 1313). It consists of 36 items to be answered on a 5-point Likert scale ranging from 1 (never/almost never) to 5 (always/almost always) based on the way the individual generally responds when confronted with a negative or unpleasant event. The questionnaire includes nine subscales, corresponding to nine cognitive strategies, with four items each. Four of the subscales are grouped as “negative cognitive ER” (self-blame, rumination, catastrophizing, and blaming others) and five subscales are grouped as “positive cognitive ER” (acceptance, positive refocusing, refocus planning, positive reappraisal, and putting into perspective). Each subscale measure is obtained by adding up scores for each of the four related items. An overall positive and negative cognitive ER score can also be obtained by finding the total of each subscale score in each category. For this study, we only considered the global positive and negative ER scores in the data analysis. The internal consistency in our sample was *α* = 0.91 for the positive scale and 0.89 for the negative scale.

#### Ecological Momentary Assessment

Using the Clinicovery web tool (https://clinicovery.com), [43]. We designed a form for adolescents to use to report the required data using their smartphones at 24 different time points. Each form consisted of a structured questionnaire, adapted from previous EMA studies on emotional and behavioral functioning e.g., [28]. The questionnaire was brief, lasting approximately one minute, and it asked the adolescents about the most negative event that they had experienced, no matter how minor, since last filling in a questionnaire.

The adolescents were first asked to indicate whether a negative event had happened or whether everything had gone fine since they had last answered the questionnaire. If a negative event had occurred, adolescents were to report the situation in which the event took place, choosing from a list of everyday social situations (friends/classmates, family, and others). Hereafter, the degree of discomfort triggered by the negative event was assessed using the following items: fear, nervousness, guilt, and sadness. Adolescents were asked to indicate the extent to which they felt each emotion or feeling during the negative event on a scale from 0 to 100 (0 = not at all, 100 = very much). The intensity of the predominant emotion (i.e., the emotion with the highest score) was selected as the indicator of the degree of discomfort for each individual during each assessment.

After indicating their momentary discomfort, adolescents were asked to choose which of two ER strategies (positive reappraisal and rumination) they had used to down-regulate their event-related negative affect (i.e., the discomfort). Momentary ER strategies were assessed via dichotomized (yes/no) variables to indicate whether an ER strategy had been used during each situation. The item related to rumination was “When you felt this bad, were you constantly thinking about how bad you felt or about the most negative aspects of the problem?” and the item related to reappraisal was “When you felt this bad, did you tell yourself that the problem wasn’t so important or try to focus on the problem in a different way so that it did not seem so bad?”.

### Procedure

Participants were accompanied by their parents or legal guardians to the university laboratory. In order to detect current adolescent psychopathological disorders, trained evaluators conducted a semi-structured interview (K-SADS-PL) with each adolescent. Their parents or legal guardians were also given the same psychopathology interview with regards to the adolescent. Afterwards, adolescents completed the self-report questionnaires (PANAS and CERQ).

A psycho-educational session was then held by the evaluators to inform the participants about reappraisal and rumination strategies. ER strategies were described in non-technical child-friendly terms e.g., [28] to ensure that adolescents would be able to identify and later report on which ER strategy they used when dealing with negative situations. During the final section, the app, which was downloaded from Google Play or the Apple Store, was installed onto the adolescents’ cell phones in order to familiarize them with the app’s interface and resolve any doubts.

At home and at pre-scheduled times, the participants were sent a notification through the Clinicovery App©, asking them to complete an electronic form (see the description in the Measures section). The adolescents were asked to report whether they had experienced negative emotions in response to a self-defined negative event that had occurred since their last completion of the electronic form. If they had, the adolescents were to fill in the form. In cases where no event qualified as aversive, adolescents were asked to report whether everything had been okay since they had last answered the questionnaire.

The data were collected over two four-day periods: from Friday after school through the following Monday evening, thus covering an equal number of school and rest days [28]. The adolescents received a notification to complete a form twice between 5 p.m. and 10 p.m. on school days (Friday and Monday) and four times between 11 a.m. and 10 p.m. on Saturday and Sunday, amounting to a total of 24 submissions. As noted above, on school days, the adolescents received the notifications in the afternoon to avoid interference with school hours. Each adolescent was paid 50 euros for participating in the study.

### Data Acquisition and Pre-processing

Since we were interested in examining how adolescents regulate their emotions during negative events, we selected only those submissions in which individuals reported their occurrence. On average, participants completed 21 out of 24 assessments (87.50%) from which 30.97% (*N* = 467) were situations in which some stressful event occurred, sparking cognitive and behavioral coping processes (i.e., the implementation of some ER strategy).

Adolescents used reappraisal in 69.60% (*N* = 325) of the assessments related to ER and rumination in 30.40% (*N* = 142) of the assessments. An individual participant’s degree of discomfort was obtained by calculating the mean score of the reported predominant emotion over all of the assessments in which the appearance of a negative event had been reported. To examine how often adolescents used each of the ER strategies, the relative use of each strategy was calculated by aggregating how often each strategy was used and dividing this number by the total of number of ER episodes reported by each individual. The relative frequencies of each type of negative situation (i.e., with family or friends) and each type of predominant emotion (i.e., anxiety-like or depression-like) were also calculated by aggregating the frequency of each type of situation or emotion and dividing these values by the total number of ER episodes reported by each individual.

Since in the preliminary analyses the correlations between the use of the ER strategies studied and the degree of discomfort (in this study discomfort values, when taking into account all of the negative episodes experienced by each participant, ranged from 2 to 100) were not statistically significant, we focused on the questionnaire from each adolescent that corresponded to the event that generated the highest level of discomfort in order to examine our second objective: assessing what factors influence the selection of a particular ER strategy (see the form description in the Measures section). Specifically, one ER event from each adolescent was selected by sampling moments containing a negative emotion of at least a moderate degree of intensity.

Once forms related to the ER event causing the greatest discomfort had been selected, adolescents’ self-reported emotions associated with the event were classified into two groups: (1) anxiety-like emotions (fear and nervousness, *n* = 30) and (2) depression-like emotions (guilt and sadness, *n* = 34). Furthermore, the type of social situation related to the self-reported negative event was also classified into two groups: (1) friend environment (*n* = 29) and (2) family environment (*n* = 22). If none of these situations appropriately described the environment in which the negative event took place, adolescents could describe the situation (20.30% of assessments). These descriptions were not included because they were not systematically reported.

### Analytical Approach

As a preliminary analysis, differences between the relative use of rumination and reappraisal of males and females were explored using *t* tests to rule out the effect of gender on the moderation analyses. Likewise, the relationship between age and the relative use of rumination and reappraisal was evaluated using bivariate Pearson correlations analysis to discard the effect of age on the moderation analyses. In addition, Pearson bivariate correlations were calculated to verify compliance with the necessary conditions of linear association between the independent variable (i.e., NA and PA) and the dependent variable (i.e., the relative use of rumination and reappraisal), as well as between the moderator (i.e., negative and positive ER style) and dependent variable [44] to establish the moderation models.

Secondly, one bootstrap moderation analysis was performed. Since the use of reappraisal did not show significant correlations with predictive and moderating variables, as will be explained later in the Results section, the corresponding moderation models were not carried out. On the other hand, since positive ER style did not correlate with the use of rumination, the corresponding moderation model was not carried out either, also explained later in the Results section. Therefore, the purpose of the bootstrap moderation analysis performed was to test both the impact of NA and the negative ER style on the use of rumination in daily life, as well as the relationship between NA and negative ER style on the use of rumination. It should be noted that due statistical correlation between PA and the use of rumination, PA was entered as a covariate in the moderation model. To estimate all path coefficients and compute associated statistics, we used the macro PROCESS version 3.4 in SPSS (SPSS Inc., Chicago, IL, USA; [45]). The number of bootstraps was set to 10,000 with confidence intervals (CIs) of 95%. In addition, to determine the effect of the independent variable on the dependent variable at different points of the moderating variable, three groups were examined for the moderating variable (cataloged as subjects with low, moderate, and high values) using the pick-a-point technique.

Thirdly, a binary logistic regression was used to assess what momentary factors influence the selection of an ER strategy, as well as the moderating role of ER style in such relationship. Since the degree of discomfort and positive ER style showed no significant correlations with the outcome variables (i.e., the use of rumination and the use of reappraisal; see Results section), these variables were not introduced into the logistic regression. In turn, as the use of reappraisal did not show a significant correlation with the predictor variables (see Results section), the corresponding regression analysis was not performed. In summary, an independent binary logistic regression could only be carried out with the selection or non-selection of rumination as the dichotomous dependent variable. In this analysis, we first entered the type of social situation that triggered the negative event (friend environment versus family environment) and the type of prevailing emotion during the negative event (anxiety-like emotions versus depression-like emotions) as binary predictors, and the overall negative ER style scores as a continuous predictor (Model 1). Negative ER style scores were converted into standardized scores to avoid multicollinearity [46] Then, in order to investigate whether the type of social situation and the type of prevailing emotion during the negative event have a greater impact on the selection of rumination for children more prone to use negative ER styles, an additional model (Model 2) with the first-order interaction terms between the overall negative ER style scores and both independent variables was fit. In the event that any of the interaction terms was statistically significant, we planned to interpret it considering the multiplicative effects approach suggested by Buis [47].

All analyses were carried out using version 25 of the IBM SPSS Statistics software package. A significance level of *p* ≤ 0.05 was adopted.

## Results

### Preliminary Analyses

The results showed that the adolescents’ relative use of rumination (boys: *M* = 0.244, *SD* = 0.298; girls: *M* = 0.256, *SD* = 0.277, *t* = − 0.184, *p* = 0.854) and reappraisal (boys: *M* = 0.567, *SD* = 0.320; girls: *M* = 0.619, *SD* = 0.293, *t* = − 0.710, *p* = 0.480) did not differ between boys and girls and were not correlated with age (Rumination: *r* = − 0.071, *p* > 0.05; Reappraisal: *r* = − 0.018, *p* > 0.05).

As initial analysis, Table 1 shows the mean, standard deviation, and pairwise zero-order correlations of all the study variables, both self-reported and those from the EMA. Overall, there were significant associations between the study variables, and most relationships were as hypothesized for the use of rumination but not for the use of reappraisal. For example, NA and negative ER style correlated positively with the use of rumination, but PA and positive ER style did not correlate positively with the use of reappraisal. In relation to the momentary variables, except for the degree of discomfort, it should be noted that the relative frequency of the type of ER strategy used (rumination/reappraisal), the type of negative situation (with friends/family) and the type of emotions experienced during the negative events (anxiety/depression) were calculated. Given that in the mobile application the adolescents could only answer one of the different alternatives, it is not surprising that, for example, in the correlation analyses the negative situations generating emotions related to anxiety correlated negatively with the negative situations generating emotions related to depression.

**Table 1:** Pearson correlations between all measures of interest and all measures’ means and standard deviation

	1	2	3	4	5	6	7	8	9	10	*M*	*SD*
1. NA	–										16.464	4.449
2. PA	− 0.185	–									22.845	2.983
3. NER style	0.667***	0.009	–								37.690	10.055
4. PER style	0.126	0.475***	0.422***	–							63.130	12.852
5. RumUse	0.271**	− 0.267**	0.394***	− 0.042	–						0.251	0.285
6. ReapUse	0.073	− 0.226*	− 0.009	0.030	− 0.119	–					0.595	0.305
7. Discomfort	− 0.013	0.028	0.007	0.119	0.120	0.121	–				52.549	27.684
8. Friends_freq	− 0.008	− 0.142	− 0.070	0.021	− 0.243**	0.125	0.120	–			0.436	0.268
9. Family_freq	0.108	0.041	0.106	0.013	0.223*	− 0.036	− 0.090	− 0.712***	–		0.377	0.287
10. Anxlike_freq	− 0.024	0.123	− 0.030	0.043	− 0.290**	0.117	− 0.297**	0.111	− 0.162	–	0.472	0.297
11. Deplike_freq	0.033	− 0.218*	− 0.034	− 0.076	0.261**	− 0.064	0.364***	− 0.078	0.214*	− 0.900***	0.447	0.293

* NA* negative affect, *PA* positive affect, *NER style* negative emotion regulation style, *PER style* positive emotion regulation style, *RumUse* the relative frequency of rumination’ use, *ReapUse* the relative frequency of reappraisal’ use, *Friends_freq* the relative frequency of negative situations with friends, *Family_freq* the relative frequency of negative situations with family, *Anxlike_freq* the relative frequency of anxiety-like emotions during negative events, *Deplike_freq* the relative frequency of depression-like emotions during negative events

*p < 0.10, **p < 0.05, ***p < 0.01

### Moderation Analyses

As described in the Methods section above, as the predictive (NA, PA) and moderator (negative ER style) variables were only correlated with the use of rumination, no moderation analyses were performed for the use of reappraisal. Moreover, since positive ER style was not correlated with the use of rumination and reappraisal, the corresponding moderation analyses were also not performed.

In the moderation model we specified NA as the predictor variable, negative ER style as the moderator variable, and the use of rumination as the outcome variable. The results indicate that the negative ER style moderated the association between NA and rumination use in daily life (see Table 2).

**Table 2:** Interaction of adolescents’ NA and NER style predicting adolescents’ rumination use in daily life

	*B*	SE	*t*	95% confidence interval	∆*R*^2^
*Regression*					0.287
NA	− 0.068	0.029	− 2.308*	[− 0.127, − 0.009]	
NER style	− 0.012	0.012	− 0.994	[− 0.035, 0.012]	
Interaction	0.002	0.001	2.239*	[0.000, 0.003]	0.054
PA	− 0.027	0.010	− 2.593*	[− 0.047, − 0.006]	

Regression analysis included PA as covariate. In addition, R^2^ of the baseline model and interaction model were included. Δ indicates a change in R^2^ due to the inclusion of the interaction term

*NA* negative affect, *NER style* negative emotion regulation style, *PA* positive affect

*p < 0.05

We further explored how the association differed in adolescents who showed low, moderate, and high negative ER style. Simple slope analyses showed that the adolescents’ NA and rumination use in daily life were significantly positively correlated when the adolescents’ negative ER style was low [*B* = − 0.028 (95% bootstrapped CI − 0.055, − 0.001), *t* = − 2.038, *p* = 0.045] but not moderate [*B* = − 0.011 (95% bootstrapped CI − 0.031, 0.008), *t* = − 1.183, *p* = 0.241] or high [*B* = 0.004 (95% bootstrapped CI − 0.016, 0.025), *t* = 0.414, *p* = 0.680].

In short, the results indicate that the impact of adolescents’ NA on rumination use in everyday life was moderated by negative ER style. Thus, adolescents with low negative ER style were more influenced by NA and reported a greater use of rumination in their daily lives than adolescents with moderate or high negative ER style.

### Logistic Regression Model

The results obtained through the logistic regression are shown in Table 3. In Model 1, we can see how the type of social situation and the predominant emotion experienced explain between 33.20% (Cox & Snell r^2^ = 0.332) and 46.6% (Nagelkerke r^2^ = 0.466) of the selection of rumination. Furthermore, this model correctly classified 88.20% of the cases. As can be seen, the results indicate that the type of social situation in which the negative event takes place explains the selection of rumination. Specifically, situations that take place in the family environment trigger a greater probability of selecting rumination as an ER strategy. Likewise, the type of emotion that prevails during a negative event explains the selection of rumination. Adolescents experiencing depression-like emotions during the negative event tend to select more rumination compared to those experiencing anxiety-like emotions. To conclude with this model, it should be noted that the model revealed a main effect of negative ER style on the rumination use. So, adolescents scoring high in negative ER style were more engaged in rumination use in their day-to-day life. Based on the data analyzed, we could say that adolescents will select more rumination when the situation in which they experience discomfort takes place in a family environment and when the emotion experienced is related to depression, as well as when they have a negative ER style. Model 2, which attempted to test for interactions between the negative ER style and both independent variables (i.e., the type of social situation and the type of prevailing emotion during the negative event) shows no significant interaction effects.

**Table 3:** Output of logistic regression models with the type of situation, the type of predominant emotion as dichotomous (dich) determinants and NER style as continuous determinant

Model and variable	*B*	Standard error	OR	95% CI of OR	
				Lower	Upper
1. Predictors					
Type of situation (family = ref.)	− 1.657	0.782	0.191*	0.041	0.883
Predominant emotion (depression-like emotions = ref.)	− 2.147	0.925	0.117*	0.019	0.716
NER style	1.236	0.478	3.442*	1.349	8.782
Constant	0.545	0.575			
2. Interactions					
Type of situation x NER style	− 1.012	1.094	0.363	0.043	3.099
Predominant emotion x NER style	− 1.597	1.097	0.202	0.024	1.740

Outcome is the selection of rumination in daily life

* OR* odds ratio, *CI* confidence interval, *NER style* negative emotion regulation style

*p < 0.05

## Discussion

The study of ER is of particular importance to research on the factors involved in the development of psychological disorders during adolescence. Traditionally, ER has been examined mainly by means of questionnaires that evaluate individuals’ habitual use of either positive or negative ER strategies, and by means of laboratory paradigms, where ER is analyzed in a controlled situation. However, research on ecological conditions is still scarce, and how adolescents regulate their emotions during everyday situations has received even less attention. To fill this gap, we examined ER in adolescents using an EMA paradigm.

In fact, an important strong point of the current study is its ecological validity. The type of social situation in which negative events occurred, the self-rated degrees of discomfort, the types of predominant emotions experienced, and the use of reappraisal and rumination were evaluated in a naturalistic context, which minimizes the problems associated with retrospective recall as it facilitates the recording of each of these variables just at the moment when the adolescent has experienced negative emotions facing negative daily events. In addition to the ecological validity of the data, the two four-day sampling period provided more comprehensive and generalizable data on ER in adolescents’ daily lives than single-set point assessments.

The first objective of the study was to investigate how temperament (PA and NA) is related to momentary ER strategies (use of reappraisal and rumination), and whether ER style (both positive and negative) plays a moderating role in this relationship. We expected that NA would predict a greater use of rumination and less use of reappraisal, and that this relationship would be moderated by a negative ER style. Conversely, we hypothesized that PA would predict a greater use of reappraisal and less use of rumination, and that this association would be moderated by a positive ER style.

With regard to rumination, as we hypothesized, adolescents scoring high in NA showed a greater use of rumination in their day-to-day life. These results are in line with prior studies showing that a heightened NA in children and adolescents is associated with a greater likelihood of engaging in the use of maladaptive strategies (e.g., rumination; [9]). Additionally, we demonstrated that the association between NA and the use of rumination was moderated by negative ER style. However, and contrary to hypotheses, the results revealed that adolescents with low negative ER style scores were more influenced by NA than those with a moderate or high negative ER style. A possible explanation for this could be that since adolescents with a low negative ER style are less involved in the use of rumination e.g., [7, 48], in these cases, the presence of a higher propensity to experience negative affect states (i.e., higher NA scores) is required compared to adolescents with a moderate/high negative ER style. However, it should be noted that the authors are surprised by the lack of interaction between NA and moderate to high negative ER style scores, given that previous work suggests that NA and dysfunctional ER style may interact to confer increased risk for emotional problems e.g., [10, 49]. Therefore, this issue should be further addressed in future ecological research.

A different pattern emerged from the relationship between PA and the use of rumination. In this case, adolescents who reported higher levels of PA were less involved in rumination during the EMA. This finding is important because, although previous studies have shown the role of PA in decreasing the use of dysfunctional strategies [10], this is among the first studies to relate PA to decreased use of rumination during daily life in adolescents. Nevertheless, the moderating function of positive ER style in this relationship could not be tested as there was no significant correlation between adolescents’ positive ER style and the use of rumination. Although adolescents with a higher predisposition to use positive ER strategies are more likely to engage less in negative strategies (e.g., rumination; [13]), results measured at the “trait” level may be independent of those measured at the “state” or “within-person” level [50, 51]. In other words, adolescents with a certain ER style can use both positive and negative ER strategies depending on the situation, and this may be reflected in our findings.

With regards to reappraisal, we were unable to examine the moderating role of ER style (both positive and negative) in the relationship between temperament and the use of reappraisal since no significant associations were found between either temperament and ER style, and the use of reappraisal in the preliminary correlation analyses. Surprisingly, and contrary to our hypothesis, correlation analyses indicate the existence of a marginally significant negative association between PA and the frequency of using reappraisal. A recent study also failed to detect a significant positive association between PA and engagement regulation (in which reappraisal is used; [10]. The authors of this study speculate that although high PA reflects a tendency to engage positively with the environment, this may not translate directly into being able to manage negative emotions with active strategies such as cognitive reappraisal. Perhaps other factors, such as emotion malleability beliefs and some cognitive control processes (e.g., working memory, set-shifting, and response inhibition) are more influential in defining adolescent propensity to using reappraisal as an ER strategy. According to Kneeland and Dovidio [52], individuals who believe that emotions are malleable are more likely to use cognitive change strategies such as reappraisal. Individual differences in working memory capacity and set-shifting costs (i.e., cognitive control processes) have also been shown to be related to differences in reappraisal capacity [53]. This idea is promoted by the interpretation that the neural regions involved in reappraisal overlap, at least in part, with those involved in cognitive control processes [54]. Moreover, also contrary to our hypotheses, no association between positive ER style and reappraisal use was found. Perhaps, as discussed above but in relation to the rumination use, these results extend the evidence found that adolescents with a certain ER style can use both positive and negative ER strategies depending on the situation, and this may be reflected in our findings.

On the other hand, the study focused on the question of whether event-related factors differentially influenced the selection of one ER strategy or another when facing a negative emotional state, and whether ER style plays a moderating role in this relationship. It was predicted that the degree of discomfort experienced during the negative event would influence the type of strategies adolescents employed. Specifically, we expected that rumination would be implemented in response to high degrees of discomfort and reappraisal in response to low degrees of discomfort.

Our results reveal that the degree of discomfort experienced by adolescents facing negative events is not associated with either the use of rumination or the use of reappraisal. Although associations between the intensity of discomfort and the use of rumination has been documented in other ecological studies [26, 27], the negative events in this study were rated by participants as moderately stressful (the mean discomfort level was 52.55 on a 0 to 100 scale), which could be the reason why the expected relationship was not found. On the other hand, the lack of association between the use of reappraisal and the degree of discomfort has been reported in the above-mentioned ecological studies. However, Silvers, Weber, Wager, and Ochsner [55]. showed that the neural basis of reappraisal varies with the intensity of the emotion-generating stimuli: reappraisal of high-intensity emotions is associated with a greater activation of the neural regions involved during reappraisal (i.e., those involved in cognitive control processes). Given the cognitive effort required to implement reappraisal, the findings of these authors are congruent with the preference for the use of reappraisal over the regulation of low-intensity stimuli [29]. It may be that, to date, ecological studies with adolescents that have linked the degree of discomfort and the use of reappraisal have not been able to detect a significant association if the degree of discomfort has been moderate, as was the case in our study.

Finally, the present study sought to examine the role played by the type of everyday situation and the type of the adolescent’s predominant emotion in the selection of one or another ER strategy when facing a negative emotional state, as well as the moderating role of ER style in this relationship. Based on our study’s findings, it appears that adolescents selected more rumination when the situation in which they experienced discomfort occurred in a family setting and when the emotion experienced was related to depression. It is likely that the relationship between negative family events and the use of rumination occurs since the family-adolescent relationship is characterized by a high frequency of conflicts [56]. Regarding the role of depression-like emotions in the selection of rumination, it is likely due to the strong association between the frequency of experiencing such emotions and adolescents’ perceived degree of discomfort. Although we have seen that the degree of discomfort in this study is not associated with the use of any of the strategies examined, these results suggest that depression-like emotions cause more discomfort than emotions related to anxiety, and that they more greatly contribute to the selection of maladaptive strategies such as rumination. In addition, as we expected, adolescents scoring high in negative ER style were more engaged in rumination use in their day-to-day life. However, no significant interaction effects were shown between negative ER style and momentary factors (i.e., the type of everyday situation and the type of the adolescent’s predominant emotion). These results probably reflect what has already been repeatedly discussed above about the possible independence between outcomes measured at the “trait” level from those measured at the “state” or “within-person” level [50, 51].

Instead, contrary to our expectations, neither the type of situation nor the type of emotion was associated with the selection of reappraisal in this study. As we have discussed above, we believe that other factors related to different types of cognitive abilities (e.g., working memory, set-shifting, and response inhibition) influence the use of reappraisal. In addition, it has already been shown that reappraisal ability (i.e., the ability to successfully perform cognitive reappraisal) and frequency are associated [53]. Since reappraisal is an adaptive strategy and individuals who use it frequently in their everyday lives report greater psychological well-being [20, 24], it is especially important that future studies examine this issue in greater depth.

Despite the contributions of this study, it has certain limitations that need to be addressed. Foremost, our study might be weakened by the small sample size owing to low participation and subsequent data losses due to the presence of some psychopathological condition or by the lack of data derived from technological errors. Moreover, we admit that given the transversal nature of most of our variables, it is difficult to conclude the analyses carried out in terms of causality; it would be interesting to complete this research with other longitudinal studies. Furthermore, we admit that our data rely exclusively on self-report measures, which creates the potential for inflated shared-source variance. However, it should be noted that although all the measures selected in this study were reported by adolescents, the use of different methods of obtaining information (i.e., self-reported questionnaires and EMA approach) allowed to reduce the problem of share-method variance. Although self-reports are considered appropriate instruments for adolescents [57], it should not be overlooked that response tendencies and social desirability may influence the data, in addition to current moods and metacognitive factors [58]. Therefore, it would be interesting to include additional measures as physiological variables associated with the use rumination and of reappraisal during the momentary ecological study. Finally, in our study we focused only on the use of rumination and reappraisal and we did not consider other ER strategies highlighted in the literature. Future research might expand the current findings by examining the role of other ER strategies (e.g., acceptance, positive refocusing, self-blame or catastrophizing) used during adolescents’ everyday life.

## Summary

This study, to the best of our knowledge, it is the first to examine the association between temperament (i.e., PA and NA), ER style, and the momentary ER strategies used in reducing negative states as well as what factors could be involved in adolescents’ selection of one ER strategy or another when facing a negative emotional state in an everyday situation. By evaluating the ER strategies used in the adolescents’ daily lives, this study provides key information to better understand the patterns of ER in adolescence, and this may have fruitful implications for preventing psychopathological problems during adolescence, thus averting more severe disorders during adulthood. Specifically, with a view to improving prevention protocols, the current findings suggest that it is essential to both reducing NA and enhancing PA for decreasing the use of maladaptive strategies (e.g., rumination) during everyday life.
